# Application of a high-resolution genetic map for chromosome-scale genome assembly and fine QTLs mapping of seed size and weight traits in castor bean

**DOI:** 10.1038/s41598-019-48492-8

**Published:** 2019-08-16

**Authors:** Anmin Yu, Fei Li, Wei Xu, Zaiqing Wang, Chao Sun, Bing Han, Yue Wang, Bo Wang, Xiaomao Cheng, Aizhong Liu

**Affiliations:** 1 Key Laboratory of Economic Plants and Biotechnology, Yunnan Key Laboratory for Wild Plant Resources, Kunming Institute of Botany, Chinese Academy of Sciences, Kunming, 650201 China; 2 Key Laboratory for Forest Resources Conservation and Utilization in the Southwest Mountains of China, Ministry of Education, Southwest Forestry University, Kunming, 650224 China; 3 University of the Chinese Academy of Sciences, Beijing, 100049 China; 4 Wuhan Genoseq Technology Co., Ltd, Wuhan, 430070 China

**Keywords:** Plant hybridization, Plant breeding, Plant evolution

## Abstract

Castor bean (*Ricinus communis* L., Euphorbiaceae) is a critical biodiesel crop and its seed derivatives have important industrial applications. Due to lack of a high-density genetic map, the breeding and genetic improvement of castor bean has been largely restricted. In this study, based on a recombinant inbred line (RIL) population consisting of 200 individuals, we generated 8,896 high-quality genomic SNP markers and constructed a high-resolution genetic map with 10 linkage groups (LGs), spanning 1,852.33 centiMorgan (cM). Based on the genetic map, 996 scaffolds from the draft reference genome were anchored onto 10 pseudo-chromosomes, covering 84.43% of the castor bean genome. Furthermore, the quality of the pseudo-chromosome scale assembly genome was confirmed via genome collinearity analysis within the castor bean genome as well as between castor bean and cassava. Our results provide new evidence that the phylogenetic position of castor bean is relatively solitary from other taxa in the Euphorbiaceae family. Based on the genetic map, we identified 16 QTLs that control seed size and weight (covering 851 candidate genes). The findings will be helpful for further research into potential new mechanisms controlling seed size and weight in castor bean. The genetic map and improved pseudo-chromosome scale genome provide crucial foundations for marker-assisted selection (MAS) of QTL governing important agronomic traits, as well as the accelerated molecular breeding of castor bean in a cost-effective pattern.

## Introduction

The spurge family (Euphorbiaceae) includes at least 6,300 species, which are widely distributed across tropical and subtropical areas. This family is composed of many important resource species, such as rubber tree (*Hevea brasiliensis* Muell. Arg.), cassava (*Manihot esculenta* Crantz), castor bean (*Ricinus communis* L.), and physic nut (*Jatropha curcas* L.). Many species in this family could accumulate unique metabolites, which are valuable resources for medicinal discovery and industrial feedstock^1^, e.g., rubber (produced by rubber tree) and ricin protein (produced by castor bean). In particular, castor bean is an important non-edible oilseed crop, whose seed oils are widely utilized in industry^2^. Due to its unique hydroxylated fatty acid (ricinoleic acid), castor oil is completely soluble in ethanol in any proportion, making it a unique feedstock for biofuel production^3,4^. Furthermore, due to its unique growth properties, including short generation time, drought hardiness, and wide adaption to different soil conditions such as barren, salted, or saline-alkali lands, castor bean is able to be planted in marginal lands, resulting in a better land use practices^5^. In recent years, castor bean has mainly been cultivated in India, Brazil, and China, for the purpose of harvesting castor oil^6^. Due to an increasing industrial demand for castor oil, there is an immediate requirement for enhanced castor seed yield through selective breeding and genetic engineering in agriculture.

According to the anthropological records, castor bean seeds have been used by humans since approximately 4000 B.C.^7^. However, molecular breeding for the genetic improvement of castor bean varieties has lagged behind that of other oilseed crops, such as rapeseeds and groundnuts. Although castor bean is a monotypic species in the genus *Ricinus*, it exhibits diverse phenotypic variations in growth habit, plant height, foliage and stem color, lateral branch nature, seed oil content, seed number, and seed size. These variable traits provide a great opportunity for germplasm selection and outcross breeding. However, a high-resolution genetic map is necessary for marker-assisted selection (MAS), because such a map could serve as a guide for the genetic improvement of varieties in breeding.

Most previous studies on genetic diversity and relatedness among castor bean germplasm were carried out using DNA markers such as AFLPs (Amplified Fragment Length polymorphisms), SSRs (Simple Sequence Repeats), EST-SSRs, and SNPs (Single Nucleotide Polymorphisms)^2,8–10^. Additionally, the availability of draft genome data of castor bean (diploid, 2n = 20) sequenced by whole genome shotgun technology serves as a vital resource for gene identification and cloning, the development of molecular markers, analysis of RNA-seq data, as well as the study of genome evolution^11–13^. The availability of the whole genome sequence for castor bean have accelerated the evolutionary history studies of castor bean and other species in the spurge family. Construction of a genetic map is a basic and powerful strategy for identification of target genes or loci highly associated with crucial agronomic traits^14^. Based on limited SSR markers, Liu *et al*.^15^ developed the first genetic linkage draft map for castor bean and facilitated research in genetics and breeding. However, the limited number and density of markers hindered the practical application of this genetic map. Due to lack of a high-resolution genetic map capable of being integrated into the draft genome sequence, it is still difficult to identify the target genes responsible for yield-related traits or to dissect the potential genetic basis of important agronomic traits. Usually, seed size and weight are important contributors to the yield increase of crop plants, so that it is critical to identify potential quantitative trait loci (QTLs) associated with seed size and weight in castor bean.

With the development of high-throughput sequencing technology, physical and genetic maps in many species could be integrated via whole genome re-sequencing (WGR) or reduced-representation sequencing^16^. In addition, genomic SNPs can be obtained using the high-throughput genotyping-by-sequencing (GBS) strategy, a low-cost and powerful approach for construction of genetic maps to connect genotypic and phenotypic variation^17^. Using GBS, the high-resolution genetic linkage maps have been constructed for many plants, e.g., rice, maize, barley, cabbage, black raspberry, sweet cherry, soybean, and banana^18–25^. The available castor bean genome at the scaffold level with 25,828 scaffolds, accounting for 92.84% of the genome size, sets a good basis for construction of a high-resolution genetic linkage map^13^. If these scaffolds can be assembled to the chromosome-level, then higher quality genomic data will be obtained, which would provide insights into the understanding of genome evolution and identification of QTLs related to important yield traits. In this study, we constructed a high-resolution castor genetic map with 10 linkage groups (LG) using the GBS method and RILs population. We compared genomic characterization among main resource plants of the spurge family, including castor bean, rubber tree, cassava, and physic nut. Furthermore, we identified QTLs that control seed size and weight in castor bean. This study not only offers a high-density genetic map which will greatly facilitate the identification of QTLs and provides genetic resources for MAS in castor bean, but also helps to explore the genome evolution of this species.

## Results

### Sequence data and SNP discovery

The RIL population used in this study was derived from the varieties ZB107 and ZB306, which show significant differences in seed size and weight traits (Fig. S1). The two parental lines were re-sequenced and yielded 8.83 Gb data for ZB107 and 8.62 Gb data for ZB306, and the sequencing depth on average was 28-fold. In total, 118,109,937 clean reads for ZB107, and 114,439,780 for ZB306, were mapped to the castor bean genome, respectively. RIL population were genotyped using GBS technology, a total of 137.5 Gb high-quality sequence data (about 97.44% bases over Q20) were obtained, with an average of 687.6 Mb data per sample. For each line, about 97% of the sequence reads were mapped to the reference genome with 10-fold coverage (see Supplementary Data S1).

In total, 1,270,605 polymorphic SNPs were detected between ZB107 and ZB306, and these SNPs were classified into four genotypes, as shown in Table 1. As the population was obtained from a cross of two parents with homozygous genotypes, only 808,191 homozygous SNPs with the segregation pattern of *aa* × *bb* were used for further analysis. SNPs with more than 25% missing data and minor allele frequency (MAF) <0.2 were filtered out. After this step, SNPs exhibiting segregation distortion ratio (p < 0.05, *X*^2^ test) were removed. Finally, a total of 15,789 SNP markers meeting these quality standards were retained to construct a genetic map.

**Table 1:** Statistic of the segregation types for SNP markers.

Marker Type	Numbers
aa × bb	808,191
nn × np	110,176
hk × hk	67,116
lm × ll	285,122
Total	1,270,605

### Construction of a high-resolution genetic map and improvement of genome assembly

**Figure 1:**
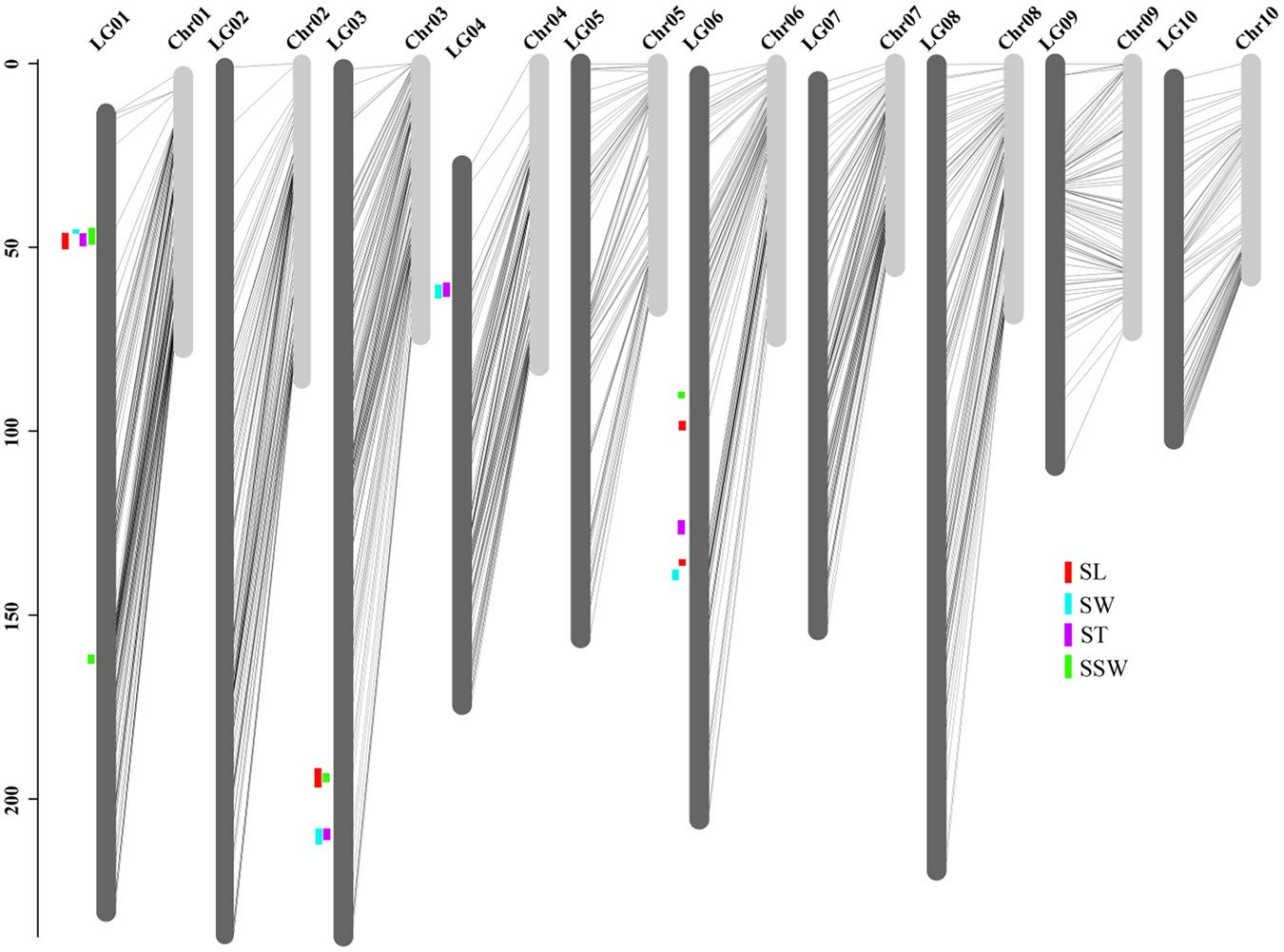
Anchored scaffolds in the assembled castor bean genome based on a genetic map and the mapped QTLs. In total, 996 assembled scaffolds (*light grey*) were anchored onto the 10 linkage groups (LG01-LG10, *grey*) using corresponding genetic markers (*bright cyan*). Positions of the mapped QTLs for seed length (SL, *red*), seed width (SW, *blue*), seed thickness (ST, *purple*), single seed weight (SSW, *green*).

Based on these SNPs, a high-density bin map was constructed for all 200 RILs, and the adjacent SNPs with the same segregation pattern were lumped as a bin marker using a Perl script. As a result, 4,317 bin markers were obtained and generated 10 LGs with a total length of 1,852.33 cM by MSTMap software (Table 2). The number of bin markers in the different LGs ranged from 238 to 540, and the length of the 10 LGs ranged from 102.35 cM to 237.71 cM. Moreover, the average intervals between two adjacent bin markers was 0.43 cM, and the average density of the genetic map was 2.33 markers per cM (Table 2). The number of markers generated in this study is 13.04 times and the density of markers is eight times more than previously reported genetic map^15^. The draft genome of castor bean was composed of 25,828 scaffolds, comprising 350.60 Mb^13^. Here, 996 scaffolds (296.01 Mb) were anchored onto 10 pseudo-chromosomes, accounting for 84.43% of the total genome size (see Fig. 1 and Table 2). Of these anchored scaffolds, 942 scaffolds were larger than 10 Kb, the longest scaffold was 4.77 Mb, while the shortest scaffold was 2.13 Kb. In total, 565 scaffolds (234.50 Mb) with two or more markers could be oriented on the pseudo-chromosomes, accounting for 66.89% of the total genome size (Supplementary Data S2). In addition, a total of 24,833 scaffolds (covering 54.59 Mb) still could not be anchored onto pseudo-chromosomes because the length of some scaffolds was too short (less than 2 Kb) to assemble, or due to the lack of SNPs on these scaffolds. Furthermore, 75.52% of the gene models (23,578 out of 31,221) were anchored onto 10 pseudo-chromosomes (Supplementary Data S3). The average length of the pseudo-chromosomes was 29.61 Mb, and the average number of scaffolds per pseudo-chromosome was 99.6. The longest pseudo-chromosome was chromosome 2 (Chr 2) with 99 scaffolds and a length of 34.84 Mb, while the shortest was Chr 10, with 75 scaffolds and a length of 23.26 Mb. The mean whole-genome recombination rate (cM/Mb) was 6.26, and these rates ranged from 3.52 on Chr 9 to 7.99 on Chr 3 (Table 2, Fig. 1). As a result, the completeness of the genome was improved based on the high-quality genetic map.

**Table 2:** Characteristics of the high-density genetic map derived from a cross between ZB107 and ZB306.

Linkage Group	Distance (cM)	No. markers^a^	No. bins^b^	Bin interval(cM)	No. anchored scaffolds^c^	Length (Mb)
LG01	235.80	1095	540	0.44	133	31.21
LG02	237.71	1143	539	0.44	99	34.84
LG03	237.61	1096	527	0.45	121	29.73
LG04	175.53	1006	452	0.39	99	32.92
LG05	159.45	922	437	0.37	71	27.58
LG06	206.82	870	438	0.47	99	30.27
LG07	157.21	841	428	0.37	108	24.93
LG08	220.54	851	441	0.50	100	27.37
LG09	119.30	597	277	0.43	91	33.91
LG10	102.35	475	238	0.43	75	23.26
Total	1852.33	8896	4317	0.43	996	296.01

^a^The number of markers.

^b^The number of bin markers.

^c^The number of anchored scaffolds.

### Comparative genome analysis of castor bean

**Figure 2:**
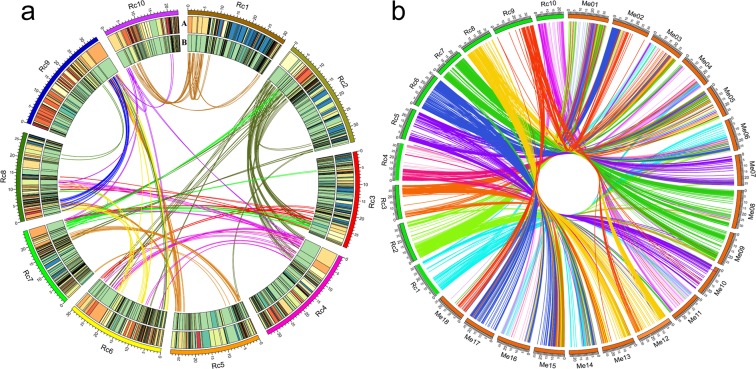
Collinearity analysis within and between castor bean genome. (**a**) Castor bean genome collinearity and the distribution of SNPs and GC content in genome. A and B indicate GC content and SNP density. (**b**) The collinear relationship between castor bean and cassava genome Rc: *Ricinus communis* L. Me: *Manihot esculenta*.

To further confirm the quality of the newly assembled castor bean genome, we performed a whole-genome collinearity analysis within the castor bean genome. A previous investigation of the evolutionary history of castor bean genome was based only on 30 pairs of scaffolds and inferred a hexaploidization event among the dicot lineage^13^, but it was impossible to analyze the intragenomic collinearity without an assembled genome at the chromosome-level. To evaluate the syntenic relationships among the ten pseudo-chromosomes in castor bean, we performed a whole-genome collinearity analysis. A total of 114 collinear blocks with ≥5 collinear genes were identified via the MCScanX software (an effective tool to analyze genome duplication and evolution)^26^. These collinear regions and their collinear relationships within the genome are illustrated in Fig. 2a. For example, a large region on Chr 2 was identified to be collinear with regions on Chr 3, 4, 6, and itself, while a small region on Chr 2 showed collinearity with regions on Chr 9 and 10 (Fig. 2a). These multiple collinear regions were distributed across different chromosomes, which strongly indicated that a whole-genome duplication (WGD) event has occurred in the castor bean genome. This may provide evidence for the presence of an ancestral polyploidization event in castor bean genome. In addition, the mean GC content was 31%, slightly lower than the GC content (32.5%) of the genome sequences, and the SNP frequency was 0.36 SNP per Kb (Fig. 2a). For interspecific collinearity analysis, we compared the synteny of castor chromosomes with those of cassava (2n = 36), whose genome has previously been assembled into chromosome-scale. In total, 1,080 collinear blocks were identified between castor bean and cassava, and blocks on every chromosome of castor bean were found to be collinear with 6 to 16 chromosomes in the cassava genome. For example, Chr 1 of the castor bean genome was aligned to 99 blocks distributed on the Chr 3, 4, 6, 11, 14, and 16 of the cassava genome. Blocks on each chromosome of the cassava genome were collinear with 4 to 9 chromosomes in the castor bean genome (Fig. 2b), e.g., cassava Chr 1 shared synteny with 114 blocks on castor bean Chr 2, 3, 4, 6, 7, 9, and 10. The results were consistent with the above hypothesis that a WGD event had occurred in castor bean, also show that there is a good synteny between the genome of castor bean and cassava.

**Figure 3:**
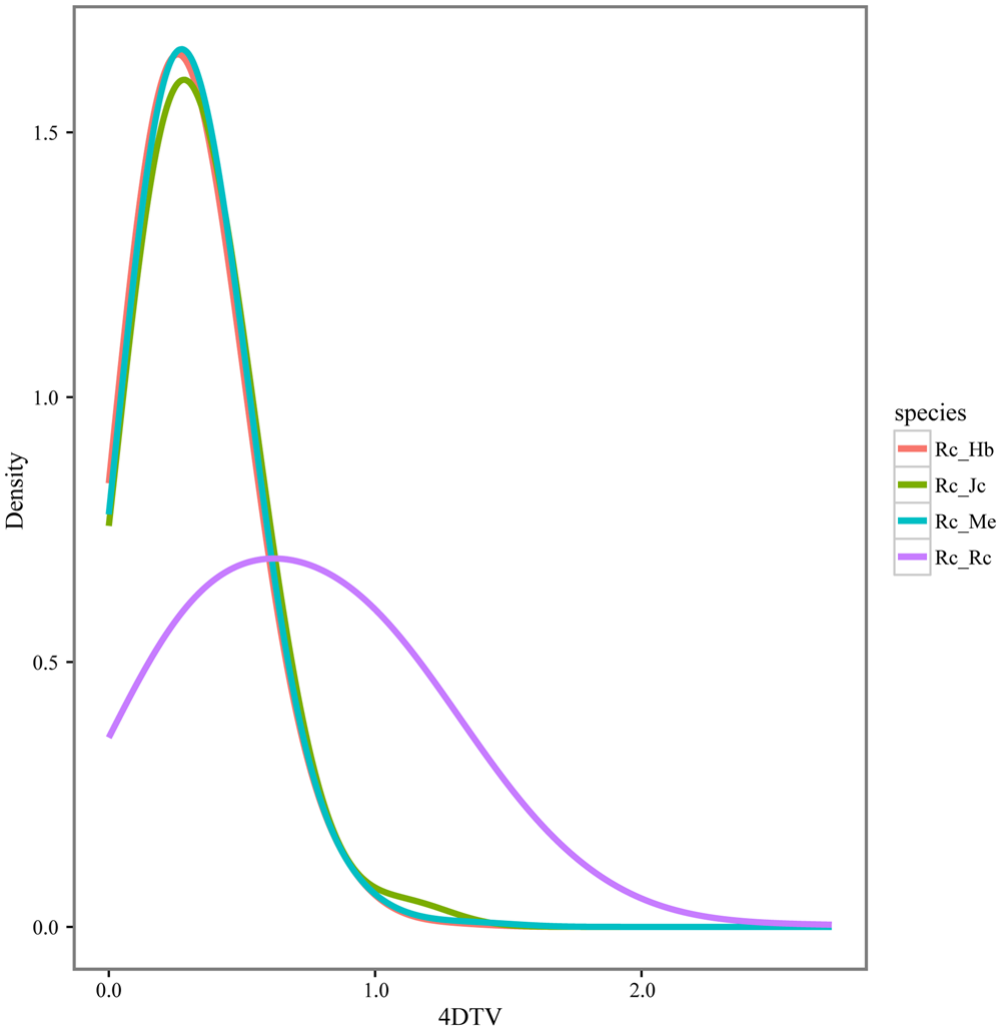
The distribution of fourfold degenerate sites (4DTV) for genes from castor bean (Rc), rubber tree (Hb), physic nut (Jc) and cassava (Me).

To reveal the possible occurrence time of WGD in the castor bean genome, the distributions of 4DTV rate among paralogous genes within the castor bean genome and orthologous genes between castor bean and physic nut, cassava, as well as rubber tree, were analyzed. The results showed that the 4DTV peaks of orthologs were at 0.2~0.3, implying that castor bean split from the three other species at roughly the same time (see Fig. 3). The 4DTV values of each pairwise paralogs peaked at 0.5 confirms that castor bean underwent an ancient WGD event before it split from these three species in Euphorbiaceae family.

### QTL mapping for seed size and weight traits

**Figure 4:**
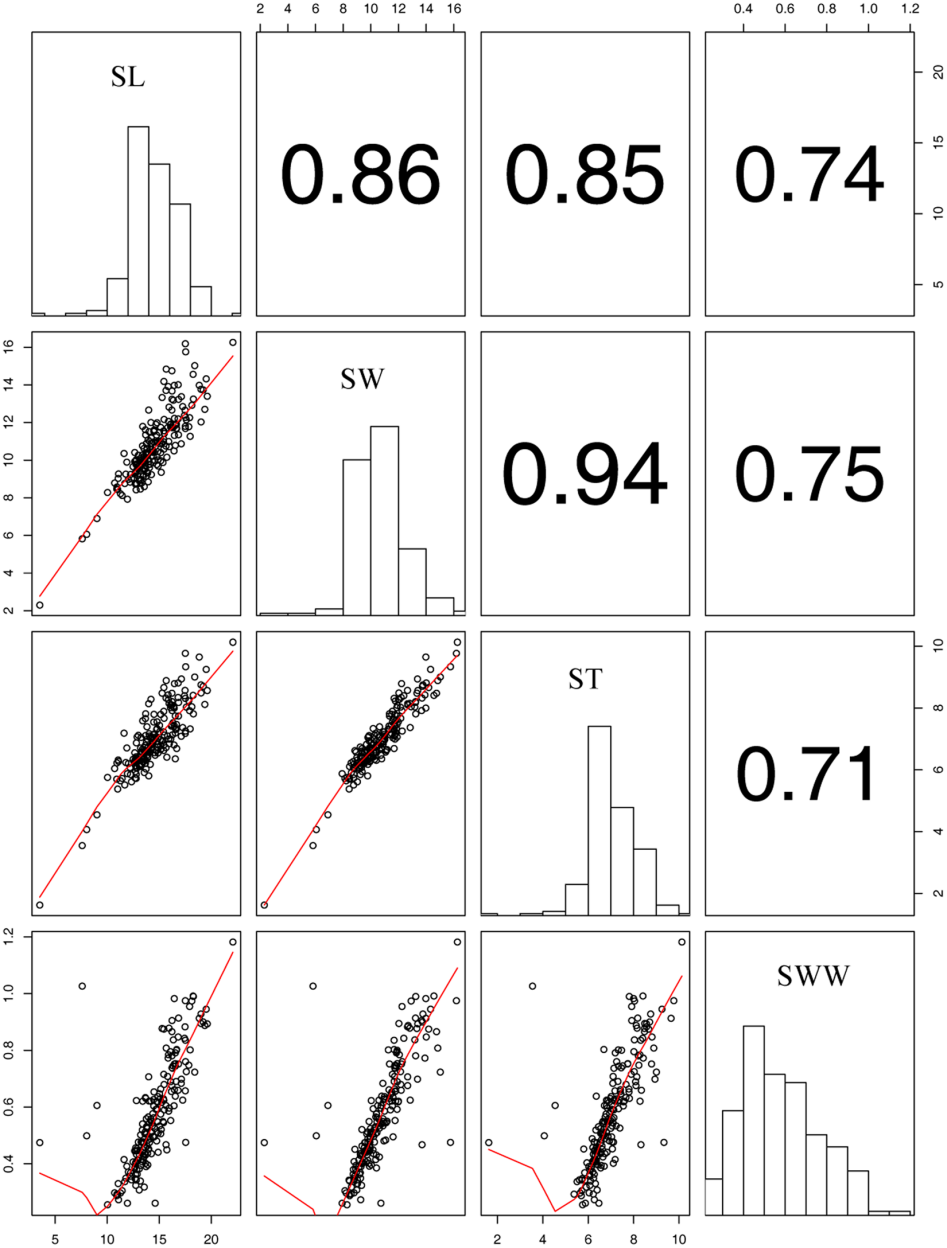
Correlations between castor bean seed size and weight phenotypes. SL, seed length; SW, seed width; ST seed thickness; SSW, single seed weight.

**Figure 5:**
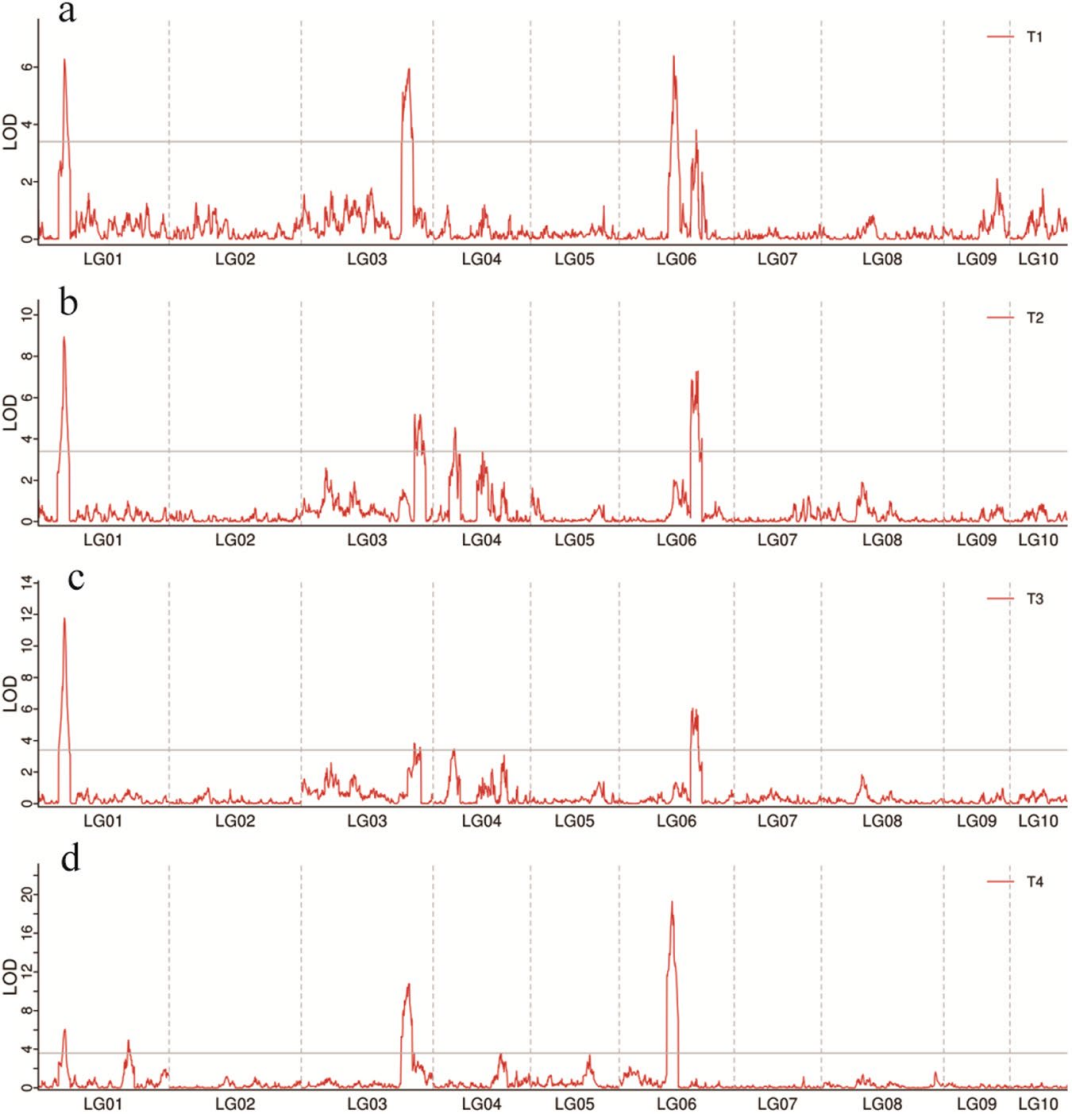
Mapping of QTL on all chromosomes for seed size and weight trait. (**a**) SL, seed length; (**b**) SW, seed width; (**c**) ST, seed thickness; (**d**) SSW, single seed weight.

To identify QTLs controlling seed yield in castor bean, we focused on seed size and weight traits in this study. The seed size and weight, including seed length (SL), seed width (SW), seed thickness (ST), and single seed weight (SSW), were measured for two parents (small-seed ZB306 and large-seed ZB107) and each line in the RIL populations. As shown in Fig. S1, ZB107 was significantly larger in SL, SW, ST and SSW, compared with ZB306. Specifically, the SL, SW, and ST were 19.80 ± 0.47, 15.43 ± 0.65, and 8.38 ± 0.31 mm in ZB107; correspondingly, they were 12.40 ± 0.77, 8.59 ± 0.07, and 6.41 ± 0.24 mm in ZB306, respectively. The SSW was 1.14 ± 0.03 g and 0.37 ± 0.03 g in ZB107 and ZB306, respectively. The two parental lines show significant phenotypic differences for the traits of seed size and weight, and they could be used to construct RIL populations to observe the corresponding phenotypic segregation among generations. In the RIL population, variations of SL, SW, and ST were 3.53~22.07 mm, 2.30~16.26 mm, and 1.62~10.13 mm, respectively, while the values of SSW ranged from 0.26 to 1.18 g (see Supplementary Fig. S1 and Fig. 4). Furthermore, we tested the correlation coefficients among SL, SW, ST, and SSW, the largest positive correlation occurred between SW and ST (r = 0.94, see Fig. 4). In total, 16 QTLs related to seed size and weight traits were identified, including 4 QTLs for each of SL, SW, ST, and SSW, respectively (Table 3, Figs 1 and 5). The phenotypic variation explained by each QTL ranged from 4.4% to 20.7%. For SL trait, *qSL6-1* has a largest effect and accounted 9.0% of the phenotypic variation, and the confidence interval was 97~99.8 cM. The major QTL for SW was *qSW1*, and it explained 11.8% of the phenotypic variation, which was located within a range of 1.3 cM, corresponding to a physical distance of 364.81 Kb on scaffold 28320 (Table 3). Notably, the region of *qSW1* was overlapped with *qSL1* and *qST1*, which could explain 8.2% and 17.2% of SL and ST phenotypic variation. These results strongly indicate that the traits related to seed size were correlated, thereby the formation of seed size might be the result of an interaction between SL, SW, and ST. In addition, two major QTLs for SSW, *qSSW3* and *qSSW6*, which explained 10.9% and 20.7% of the phenotypic variation, spanned a genetic distance of 2.6 and 1.6 cM with high LOD scores of 10.78 and 19.31, respectively.

**Table 3:** QTL associated with SL, SW, ST and SSW traits.

Trait^a^	QTL	LG^b^	Position(cM)	Interval(cM)^c^	LOD	ADD^d^	R^2e^
SL	qSL1	01	47.11	46.2–50.3	6.28	0.67	8.2%
SL	qSL3	03	194.41	191–196.3	5.95	−0.67	7.7%
SL	qSL6-1	06	98.01	97–99.8	6.40	−0.93	9.0%
SL	qSL6-2	06	138.51	138.4–139.8	3.81	−0.69	5.1%
SW	qSW1	01	46.41	45.5–46.8	8.94	0.6	11.8%
SW	qSW3	03	214.31	212.7–216.7	5.17	−0.5	6.5%
SW	qSW4	04	39.51	37.4–40.5	4.54	−0.4	5.7%
SW	qSW6	06	141.71	140.4–143.1	7.28	−0.60	9.1%
ST	qST1	01	47.11	46.2–49	11.77	0.45	17.2%
ST	qST3	03	213.91	212.7–215.4	3.58	−0.25	4.7%
ST	qST4	04	37.91	37.1–40.3	3.32	−0.23	4.4%
ST	qST6	06	132.01	130.6–133.6	6.05	−0.33	8.0%
SSW	qSSW1-1	01	48.11	45.3–49.6	6.07	0.06	6.2%
SSW	qSSW1-2	01	162.71	161.8–163.6	4.95	0.05	4.6%
SSW	qSSW3	03	194.41	192.5–195.1	10.78	−0.07	10.9%
SSW	qSSW6	06	94.81	94.3–95.9	19.31	−0.09	20.7%

^a^Traits related to seed length (SL), seed width (SW), seed thickness (ST), and single seed weight (SSW) are listed.

^b^LG, linkage group.

^c^the 95% confidence interval for QTL location.

^d^ADD, the additive effect value.

^e^R^2^, the contribution rate of the locus to the phenotype.

### Identification of potential candidate genes

To detect the potential genes controlling seed size and weight traits, we further dissected 851 candidate genes within all physical regions of these identified QTLs according to the castor bean reference genome and gene annotation (see Supplementary Data S4). We conducted a KEGG analysis to explore the potential functions of these candidate genes. As shown in Supplementary Fig. S2, the top five enriched pathways were involved in “Translation”, “Folding, sorting and degradation”, “Amino acid metabolism”, “Carbohydrate metabolism”, and “Lipid metabolism”. A large number of detected genes were functionally involved in metabolism processes or were classified into transcription factor, ubiquitin-mediated proteolysis, and plant hormone signal transduction (also see Supplementary Data S4). We compared these identified genes from all QTLs and found that 63 genes were co-localized among the four traits (SL, SW, ST and SSW) (Supplementary Fig. S3). These genes were functionally involved in various biological processes including protein metabolism, phenylpropanoid biosynthesis (such as the gene 28320.m001136), and hormone signal transduction. Sixty-six genes were co-localized to the same genomic regions among the three traits (SL, SW, and ST) related to seed size, including two genes encoding protein precursor of brassinosteroid-regulated xyloglucan endotransglucosylase/hydrolase (29993.m001055 and 29993.m001056). We noted that 34, 185, 45, and 13 genes were separately detected from the traits of SL, SW, ST, and SSW, respectively, including the gene from *qSW3*, 29726.m003935, encodes TRANSPORT INHIBITOR RESPONSE 1 protein. In addition, we noted that six genes encoding peroxidases (28320.m001136, 29726.m003966, 29726.m003965, 29726.m004037, 29889.m003322, and 30147.m014131) were identified from QTLs, which might be involved in regulating the development of seed coat in castor bean.

## Discussion

Genetic maps have long been used to accelerate the molecular breeding of many species. The construction of a high-resolution genetic map of castor bean would greatly speed up the breeding process by maximizing our ability to identify the genomic regions associated with important agronomic traits. The linkage map of castor bean reported previously by Liu *et al*. consisted of only 331 SSR markers, spanning 1164.73 cM, with an average interval distance of 3.63 cM between markers^27^. In this study, we constructed a high-quality SNP-based map using 4,317 non-redundant bin markers with a smaller interval distance of 0.43 cM between markers, covering 1,852.33 cM. The high abundant SNP markers discovered by GBS highly improved the marker density of current genetic map. GBS is a simplified sequencing technique, which was useful for large-scale SNPs discovery, genetic map construction, QTL mapping of important traits, as well as improvement of draft genome assembly in many other organisms^27^. Furthermore, the type of mapping population was an essential factor for genetic map construction and QTL mapping. Compared with the previous genetic map generated by Liu *et al*. using an F_2_ population, our genetic map was constructed based on a permanent RIL population. It has been widely accepted that an RIL population decreases heterozygosity and increases the frequency of homozygous loci, making dominant markers more informative^28–30^. To date, our map is the densest genetic linkage map with the highest density SNP markers, which will provide a basis for MAS and genomic studies of castor bean.

One of the main purposes of this genetic map is to update genome assembly of castor bean. The quality and density of genetic maps are important factors in guiding the anchoring of scaffolds onto pseudo-chromosomes^31^. This method has been applied in many species; for example, a genetic linkage map of the soybean was of great help to anchor and orient more than 97% of the whole genome sequence^24^. Similarly, the sesame genome assembly was substantially updated with a high-density genetic map^32,33^. Based on the RIL population and genomic SNP markers, we assembled 996 scaffolds from the castor bean reference genome into 10 pseudo-chromosomes, covering 84.43% of genomic sequenced data. Collinearity analyses not only indicated that we produced an updated high-quality of assembly genome, but also revealed that the castor bean genome had high collinearity with the cassava genome. Furthermore, collinearity and 4DTV analyses reveal that a WGD has occurred during castor bean genome evolution, and this event might be shared by castor bean and other members in the Euphorbiaceae family. These results support Chan *et al*.’s hypothesis that hexaploidization arose during castor bean evolution^13^, but we are not certain the event occurred ubiquitously during the evolutionary processes of the core eudicots or not^34^. Collectively, these results confirm the hypothesis that the phylogenetic position of castor bean is relatively solitary from other taxa in the Euphorbiaceae family.

The high-density linkage map and newly generated genome at the pseudo-chromosome level provided precise locations for mapping QTLs associated with seed size and weight traits in castor bean. A total of 16 putative QTL regions were identified for controlling seed size and weight, and these QTLs were distributed on four Chromosomes (Chr 1, 3, 4, and 6). QTL regions for SL, SW and ST traits were co-localized on Chr 1, 3 and 4, suggesting that these regions are critical for controlling the seed size, and the genetic bases are correlated among the different traits related to seed size variation. As previously noted, GS5 is an important QTL in rice, regulating both grain width and weight^35^. Furthermore, these QTL regions associated with two or more traits were pleiotropic effects or close linkage, so that the markers within these QTLs may have greater potential use in future molecular breeding and genetic improvement of castor bean.

All QTL regions cover 851 candidate genes, and these promising genes are functionally involved in diverse functional categories, such as metabolism, transcription factor, ubiquitin-mediated proteolysis, and plant hormone signal transduction. Generally, for mature seeds with endosperm (such as rice and maize), seed size is closely related to the formation of seed coat, while seed weight strongly depends on the development of the endosperm^36^. The castor bean is a typical dicot seed with a persistent endosperm, while the development of the seed coat acts as an upper limit for the final seed size^37^. Lignification of secondary cell walls is critical in determining seed size during seed coat development. Correspondingly, cellulose, xylem, and lignin are synthesized during cell wall lignification^38^. Previous studies have shown that peroxidases not only directly participated in the process of lignin polymerization but also played a critical role in regulation of cell elongation and the maintenance of auxin levels^39^. In this study, six peroxidase genes were identified from QTLs, implying that the seed size of castor bean is probably determined by cell wall lignification and seed coat development. Recently, we have verified that these peroxidase genes are most likely the targeted genes responsible for regulating cell wall lignification of the seed coat in castor bean^40^. In addition, previous studies have shown that brassinosteroid plays a critical role in controlling cell expansion by changing the properties of cell walls (such as loosening and rearranging the cell wall) during seed coat development^39^. Two brassinosteroid-regulated xyloglucan endotransglucosylase/hydrolase genes (29993.m001055 and 29993.m001056) were identified from all three QTLs (*qSL6-2*, *qST6* and *qSW6*), suggesting that brassinosteroid might be involved in the regulation of seed coat development. In addition, many studies have shown that auxin is critical in driving seed coat development by regulating cell division^40,41^. The identification of the 29726.m003935 gene encoding an auxin receptor protein TIR1 from *qSW3*, indicates that auxin signal transduction may also be important in regulating the formation of seed size. Unsurprisingly, many identified genes from SSW traits are functionally involved in metabolic processes; in particular, in the regulatory processes of starch and sucrose metabolism, as well as storage material accumulation. Furthermore, these identified genes provide great resources to dissect the molecular mechanisms underlying the endosperm development among castor bean varieties with variable traits in seed weight. In addition, we noted that many of the identified genes were considered as conserved proteins due to the low quality of gene annotation in the available reference genome of castor bean. Collectively, these candidate genes provide directional clues to detect potential major-effect genes responsible for seed size and weight traits in castor bean. Lastly, this study also lays a solid foundation for further dissection of the genetic mechanisms underlying the formation of seed size and weight in typical endospermic seeds of dicot plants.

## Conclusion

This study represents the first high-resolution genetic map with 10 linkage groups for castor bean, containing 8,896 SNP markers with a total genetic length of 1,852.33 cM. This genetic map was used to guide 996 genome assembly scaffolds anchoring onto the pseudo-chromosome level, accounting for 84.43% of the whole genome. Based on the newly assembled genome, collinearity and comparative genome analyses provide new evidence in understanding genomic variation during the evolution of castor bean genome. We detected 16 QTLs related to seed size and weight traits, covering 851 candidate genes. To date, this work is the first high-resolution genetic map and gene-level QTL based on GBS and RIL population in castor bean, which will greatly facilitate the identification of quantitative trait loci and molecular breeding research in the future. Furthermore, this map must be a major step in speeding up the molecular breeding and genetic improvement of castor bean.

## Materials and Methods

### Plant materials

Two castor bean inbred lines, ZB306 (with small seeds) and ZB107 (with large seeds) kindly provided by Shandong Zibo Academy of Agriculture Sciences, China, were used as parental lines to generate an RIL population by the single seed descent method. The two parents exhibit drastic morphological differences in seed size, seed weight, oil content, stem color, plant height, inter node length, panicle number, and fruit number. F_1_ hybrids were produced by crossing ZB306 and ZB107, and then selfed to generate the F_2_ population with segregating phenotypes of the traits mentioned above. F_2_ individual plants were selfed until the F_4_ generation from 2011 to 2015, and a final set of 200 F_4_ RILs was obtained. Two parents and the F_4_ population were grown under field conditions in the experiment field of the Kunming Institute of Botany of the Chinese Academy of Sciences (CAS) in Kunming, Yunnan Province, China, following a randomized complete block design with three replicates in 2015. Each plot contained 6 plants per row, with 1 m row spacing and 1 m plant spacing. The seeds from each plot were individually harvested, cleaned, and dried at room temperature. To determine seed size and shape, seed length, width, and thickness of ten seeds from three plants in each replicate were measured with calipers, and the average of these seeds was used to represent the trait measurement. We measured the weight of ten seeds and then converted it to single seed weight (SSW). The frequency distributions of seed size and weight traits for all 200 lines and correlation analysis of the seed size and weight traits were performed using R package software (https://www.r-project.org/).

### DNA extraction and Genotyping-by-sequencing

DNA was isolated from the 2-month-old castor bean plants of each of the 200 RILs. DNA extraction was carried out using the plant genomic DNA extraction Kit (TIANGEN, Beijing, China) following the manufacturer’s instructions. RNase A was then added to digest RNA. The quality and concentrations of DNA were detected using a NanoDrop 2000 (Thermo Fisher Scientific, USA), while DNA integrity was examined by electrophoresis on 1% agarose gels.

To prepare the reduced representation libraries for sequencing, the GBS protocol was carried out according to the method reported by Elshire *et al*.^17^. In brief, the genomic DNA was first digested by restriction enzymes. In this case, *Eco*RI and *Mse*I were selected to efficiently reduce genome complexity. The barcode adapters and common adapters were then linked to the sequence ends of the fragmented DNA samples. PCR amplification and fragment selection was performed. Libraries were then sequenced by Illumina HiSeq3000 platform, which generated 150 bp paired-end reads.

### Sequence analysis and SNP discovery

GBS sequencing reads of each individual RIL were sorted according to the barcoded adapter. Reads were filtered with unidentified nucleotides (N) more than 10%, or trimmed with >50% bases having a phred quality <5. Afterward, reads with more than ten nucleotides aligned to the adapter sequence or reads containing the *Eco*RI and *Mse*I enzyme sequences were removed before proceeding to further analysis. The filtered reads from the two parents (the whole genomes of ZB306 and ZB107 had been re-sequenced in our previous study) and 200 offspring were mapped to the reference genome (http://castorbean.jcvi.org) using the BWA software (settings: mem -t 4 -k 32 -M -R)^42^, while reads mapped on the multi-position of genome were removed. After this step, the alignment files were converted to BAM file formats by SAMtools software (settings: -bS -t)^43^. Read grouping and removal of PCR duplications were done using Picard (https://sourceforge.net/projects/picard/). Variants and genotypes were called for all the samples using the HaplotypeCaller function in GATK^44^. The parental genotypes were analyzed by inspecting both homozygous and heterozygous SNP alleles, and only the homozygous SNPs with at least five reads coverage were used to detect the genotypes of the RIL population.

### Construction of linkage map and improvement of genome assembly

Before linkage map analysis, the SNP markers with missing data >25% were discarded and segregation distortion was tested with a Chi-square test (*p* < 0.05). For linkage analysis, the adjacent markers with same genotype were regarded as a bin marker using an in-house Perl script. To construct a genetic linkage map, the bin markers were used to form a skeleton bin of castor bean linkage groups using MSTMap software (http://alumni.cs.ucr.edu/yonghui/mstmap.html) with a minimum logarithm of minimum odds (LOD) score threshold of 10.0. Recombination values between markers were converted to map distances in centiMorgan (cM) using the Kosambi mapping function. A linage map was drawn using a Perl SVG module^45^. To anchor these scaffolds onto pseudo-chromosomes according to the genetic map, the SNP markers of the genetic map were used to BLAST with the draft genome of castor bean at an E-value cutoff of 10^−40^. Unique scaffold sequences were aligned with the marker sequences based on the criteria of >95% identity, and the location of scaffolds was decided by the corresponding marker locus.

### Comparative genomic analysis

An all-against-all BLASTP search was performed with an *e*-value cutoff of 1*e*-5 between protein sequences from the castor bean genome, as well as castor bean and cassava genomes. The MCScanX software was used to identify chromosome collinearity within castor bean genome, as well as between castor bean and cassava genomes^26^. Meanwhile, protein sequences from castor bean, physic nut, rubber tree and cassava were performed via an all-against-all comparison using BLASTP with an *e*-value cutoff of 1*e*-5. Gene clusters of the four species were identified using OrthoMCL v2.0.9, with a default inflation value of 1.5^46^. Meanwhile, the paralogous gene pairs within castor bean, and the orthologous gene pairs between castor bean and other Euphorbiaceae members were identified. ClustalW with default parameters was used to align protein sequences of paralogous and orthologous gene pairs. To estimate the duplication event, the fourfold synonymous third-codon transversion (4DTV) rate was calculated according to Tang’s method using in-house Perl scripts^47^. Collinearity blocks, SNP density, and GC content of each of the pseudo-chromosomes of castor bean were plotted and visualized by Circos (version 0.67–7)^48^.

### QTL and candidate gene analysis

QTLs for seed size and weight traits were detected by Windows QTL Cartographer 2.5 software using the composite interval mapping (CIM)^49^. The LOD threshold was determined using 1000 permutations with a threshold value of p < 0.05. The additive effect of QTL and the proportion of the phenotypic variance explained by QTL (R^2^) were estimated. QTL names were designated according to the trait and linkage group locations. The potential candidate genes were collected from the refined QTL regions and their functional annotation were retrieved using gene models from the castor bean genome, and the regions were limited by the SNP markers on the linkage map and reference genome. The Kyoto Encyclopedia of Genes and Genomes (KEGG) pathway enrichment analysis was performed to detect potential pathway of these candidate genes.

## Supplementary information

Supplementary Information

Dataset 1

Dataset 2

Dataset 3

Dataset 4

## Data Availability

The raw sequence data of GBS was submitted to the Sequence Read Archive (SRA) under accession PRJNA530041. Most of the data sets generated during this study were included in this published article and in supplementary information files.
