# Supplementary material for: Application of a high-resolution genetic map for chromosome-scale genome assembly and fine QTLs mapping of seed size and weight traits in castor bean
Source: Sci Rep. 2019 Aug 16;9:11950. doi: 10.1038/s41598-019-48492-8 (PMC6697702; doi:10.1038/s41598-019-48492-8)
Supplement: Supplementary file 1 — Supplementary Information [file 41598_2019_48492_MOESM1_ESM.docx]

**Application of** **a** **high-resolution genetic map for chromosome-scale genome assembly and fine QTLs mapping of seed size and weight traits in castor bean**

Anmin Yu^1,3^, Fei Li^1^, Wei Xu^1^, Zaiqing Wang^1,3^, Chao Sun^1^, Bing Han^1,3^, Yue Wang^1^, Bo Wang^4^, Xiaomao Cheng^2^, Aizhong Liu^2*^

^1^Key Laboratory of Economic Plants and Biotechnology, Yunnan Key Laboratory for Wild Plant Resources, Kunming Institute of Botany, Chinese Academy of Sciences, Kunming 650201, China

^2^Key Laboratory for Forest Resources Conservation and Utilization in the Southwest Mountains of China, Ministry of Education, Southwest Forestry University, Kunming 650224, China

^3^University of the Chinese Academy of Sciences, Beijing 100049, China

^4^Wuhan Genoseq Technology Co., Ltd, Wuhan 430070, China


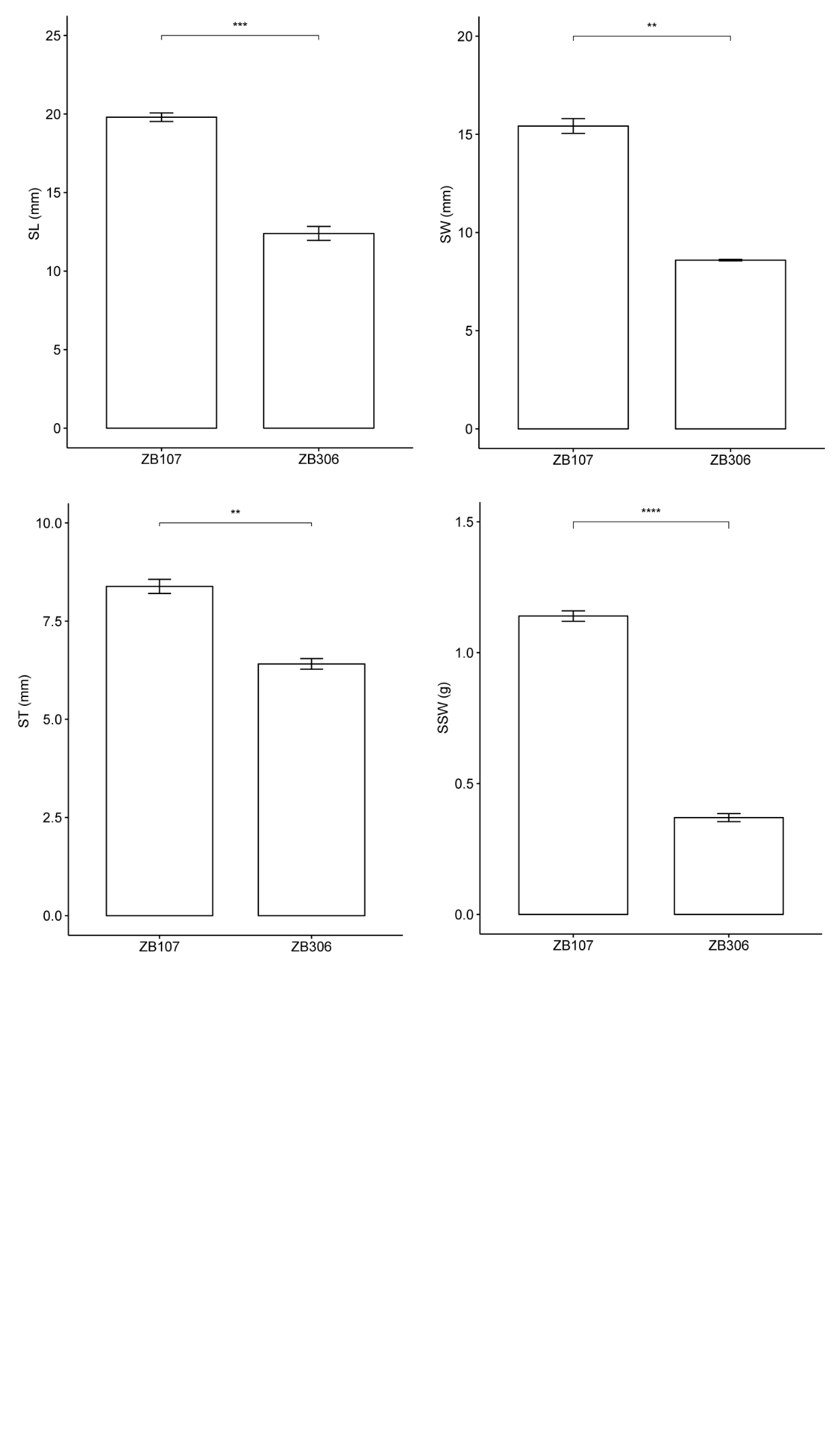


**Figure S1** Variations in seed size and weight traits in the two parents. The *x*-*axis* shows the castor bean varieties ZB107 and ZB306. The *y*-*axis* shows the phenotypic variation of SL (seed length), SW (seed width), ST (seed thickness), SSW (single seed weight). Values are reported as mean ± SD (n =10), ** P< 0.01, *** P< 0.001, **** P< 0.0001 (Student’s test).


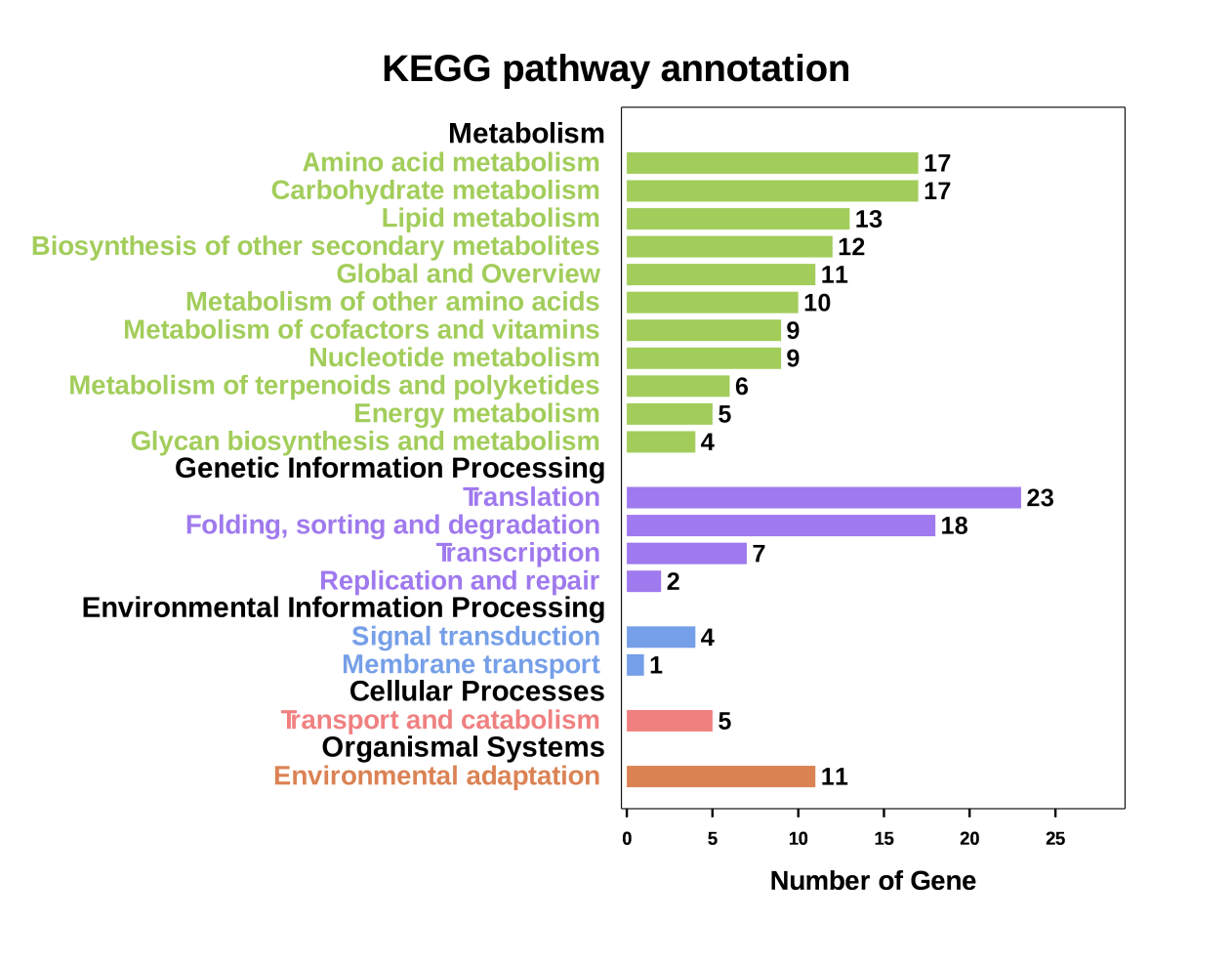


**Figure S2** KEGG pathway annotation of the genes related seed size and weight traits.


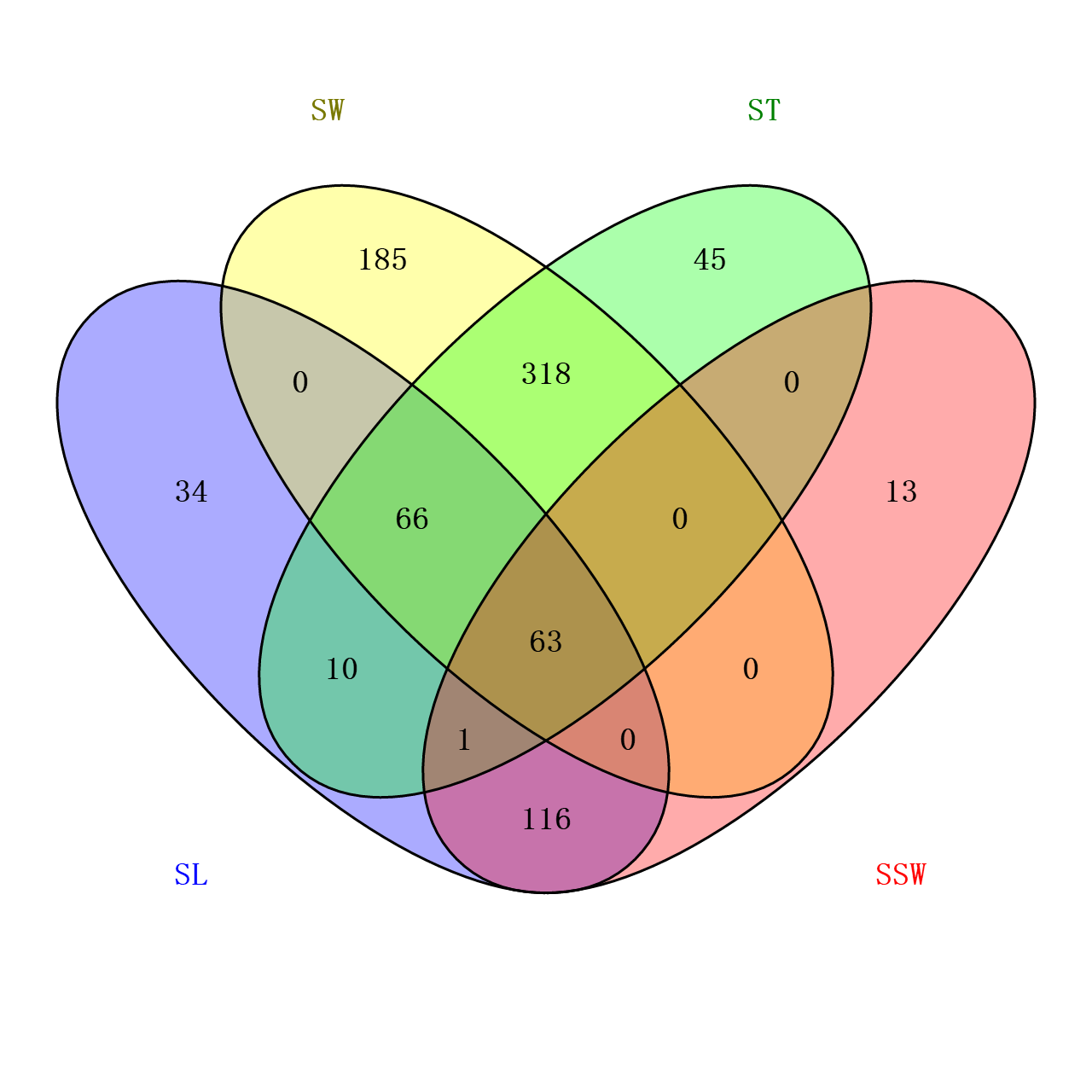


**Fig****ure S3** Venn diagrams of candidate genes located in the traits of SL, SW, ST and SSW.
